# Norwegian Men and Women Value Similar Mate Traits in Short-Term Relationships

**DOI:** 10.1177/1474704920979623

**Published:** 2020-12-29

**Authors:** Mehmet Mehmetoglu, Ilmari Määttänen

**Affiliations:** 1 Department of Psychology, Norwegian University of Science and Technology, Trondheim, Norway; 2 Department of Psychology and Logopedics, Faculty of Medicine, University of Helsinki, Finland

**Keywords:** mate preference, sex differences, sexual selection, long-term mating, short-term mating

## Abstract

Previous research has provided evidence that females are generally the more selective sex in humans. Moreover, both sexes have been found to be more selective in long-term mating compared to short-term mating. In this study, we have examined the effects of sex, mating strategy (preferred relationship length) and their interaction on mate preferences (i.e., mate selection criteria) in an egalitarian Nordic society, namely Norway. The study sample consisted of 1,000 individuals, 417 of whom were male and 583 female respondents. According to our findings, men were more selective in physical appearance, whereas women were more selective in all the other mate preferences (e.g., understanding, dominant, kind, intellectual etc.). The respondents that were seeking short-term relationships had higher preference for physical appearance, humorousness and sociability. On the other hand, the respondents that were seeking long-term relationships were more selective in most of the other mate preferences (i.e., understanding, kind, cultivated, domestic, reliable, and similar). Interestingly, no interaction effect was found between sex and mating strategy in that differences between long-term and short-term seekers in mate preferences did not change depending on sex. This suggests that men and women value the same traits in short-term relationships.

## Introduction

There is an ongoing debate about the relative importance of mate preferences (i.e., mate selection criteria) depending on sex in short-term versus long-term relationships. According to theory on sexual selection, the sex that invests more energy and other resources to the offspring is typically the more selective sex (Trivers, 1972). Among most mammals, females are typically the more selective sex, as their minimum effort for producing offspring (i.e., being pregnant and giving birth) is much higher than minimum effort for males (i.e., producing sperm). This is true for humans as well: women are the more selective sex for most traits with the exception of physical attractiveness, in which men are typically more selective (Buss & Schmitt, 1993; Castro & Lopes, 2011; Regan et al., 2000). Monogamous species have often small sex differences in mate preferences. Since humans are mostly monogamous, it is not surprising that some studies have not found sex differences in selectivity in humans (Mogilski et al., 2019).

Several attempts to explain mate preferences among humans have been made. Perhaps the most influential of them is Sexual Strategies Theory (SST) put forward by Buss and Schmitt (1993). Mate choice, according to Sexual Strategies Theory, is highly sensitive to the temporal context of short-term versus long-term partnerships. Based on different minimum parental investment of different sexes, men are predicted to prefer more sexual partners and variety, i.e. more short-term mates. This has been replicated by several studies (Kurzban & Weeden, 2005; Oliver & Hyde, 1993; Schmitt et al., 2001; Shackelford et al., 2004). Thus, short-term relationship seeking is believed to be much more common among men than women. Although it is generally known that both sex and mating strategy influence mate choice behavior, there is still surprisingly little detailed knowledge about specific mate preferences or criteria sought by short- and long-term relationship seekers in the two different sexes. The ambiguity remains because interaction-effects have not been explicitly studied in many of the relevant studies. A competing hypothesis for SST is Attachment Fertility Theory (AFT), which postulates that both of the sexes display similar preferences in contexts where the requirements for their parental investment are similar (Miller et al., 2005). At least one study did not find a relationship length-sex interaction in mate preferences and it has accordingly been noted that this finding acts as evidence against the SST (Pedersen et al., 2014). The study found that men do not “lower” their standards for short-term mating more than women do, which is a conflicting finding against SST.

Overall, in spite of some criticism, the rational for SST seems plausible at first glance and it has inspired a lot of research. For women, according to SST, the predicted underlying primary goal in short-term relationships is different from men: they need to secure possible resources in the short term, and to assess the long-term prospects of a mate. Women also need to pay special attention to the “genetic quality” of the partner, which is sometimes used synonymously with attractive appearance (Asendorph et al., 2011; Buss & Schmitt, 1993). For men, on the other hand, the greatest limitation for short-term mates is their access to women, as men do not have an “unlimited” number of willing mating partners. Men may indeed differ in their mating strategies (Gangestad & Simpson, 2000). In the discussion on the topic of men’s mate preference, it is often not stressed enough that other preferences can only be considered after they have succeeded in finding willing partners. The end result may be a compromise between women’s and men’s mating preferences (Jonason et al., 2009). Most of the studies have found men place higher preference for physical appearance than women, which may sound counterintuitive, given the aforementioned logic (Buss & Schmitt, 1993; Castro & Lopes, 2011; Regan et al., 2000). Women’s preference for physical attractiveness may lead to “sexy sons” rather than overall increased viability of the offspring, i.e. “good genes” (Prokop et al., 2012).

There is evidence that human women have a higher preference for resources and for traits that may influence the accumulation of resources than men do (Ong & Wang, 2015). Buss and Schmitt (1993) tested multiple hypotheses related to sexual selection in men and women. They found a clear difference between sexes in the preference for physical attractiveness: men regarded physical attractiveness as more important than women in several different cultures. Good financial prospects in a partner were more important for women. Perhaps surprisingly, in a study of speed dating, facial attractiveness was the most important trait that affected the likelihood of being chosen among both sexes (Asendorph et al., 2011). In the same study, financial prospects were more important for long-term relationships than short-term relationship.

Physical attractiveness has also been found to be the most significant predictor of mate preference in several other dating-based studies (Kurzban & Weeden, 2005; Luo & Zhang, 2009) and in a meta-analysis (Eastwick et al., 2014). According to Gustavsson and colleagues (2008), in a study of online dating in Sweden, women preferred men who possessed the ability to acquire resources, and men advertised this ability. On the other hand, there was no sex difference in demanding or advertising good appearance (Gustavsson et al., 2008). Shackelford and colleagues (2005) found four factor dimensions from an 18-item questionnaire across cultures. Women had a higher preference for resources, dependability and intelligence, and men had a higher preference for good looks, health and willingness to have children. In a study of Brazilian undergraduate students, men had a higher preference for physical attractiveness, but the sex difference was smaller in short-term mate seekers. In general, women were more selective (Castro & Lopes, 2011). On the other hand, women were somewhat less selective in terms of resources when the preference was contrasted with “good looks” in short-term partners (Li & Kenrick, 2006).

Several studies have also been paying attention to relationship length. Stewart and colleagues (2000) found that US students had higher standards for long-term mates than short-term ones, and that men preferred more appearance and reproductive value-related traits, whereas women preferred “resource acquisition ability”-related traits. According to a study by Li and Kenrick (2006), “the sexes are similarly selective for long-term relationships, whereas women are more selective regarding short-term relationships” (p. 483). The study also found a significant interaction-effect in which the sexes were more similar in their preferences for short- versus long-term mates: both sexes prioritized physical attractiveness for short-term mates whereas women were less selective for long-term mates’ appearance (Li & Kenrick, 2006).

According to Regan and colleagues (2000), women valued social status and resources more than men did, and men valued physical attractiveness and sexual desirability (which includes sexy appearance and being sexually passionate) more than women did. In addition, both sexes valued sexual desirability more when it comes to short-term mates. A significant sex-relationship length interaction was found, in which women displayed a higher preference for partner’s sexual passion and desire for short-term partner than long-term partner, whereas there was no such difference among men.

Jonason and colleagues (2013) noted that human mate preferences are often studied by single-item measures and no factor analysis is utilized. An outline of the analysis strategy utilizing factor analysis was suggested by Bond (1988). Preference studies have, according to Jonason and colleagues (2013), too often concentrated on long-term partner preferences. Their study did not, however, involve interaction analyses but analyzed different sexes and their short- and long-term partner preferences separately. Jonason and Antoon (2019) also studied several interactions involving the level of education and personality in preferred partners. These analyses did nonetheless not involve interactions comparable to those of the current study.

According to the literature reviewed above, the issue of relationship length and sex interaction has not been very clearly treated in much of the previous research. In some studies, where such interaction could have been assessed, the interaction analysis and suitable statistical analyses are lacking (Stewart et al., 2000). There was an interesting finding about a lack of sex-relationship length interaction, which was correctly pointed out to be evidence against the prevailing Sexual Strategies Theory (Pedersen et al., 2014). Overall, however, it seems that not much in-depth discussion surrounding the interaction topic has been made. In addition, what makes the current study different from others is that the data provided here is from Norway, which could be described as one of the most egalitarian countries in the world. Incidentally, Norway was ranked as the second most egalitarian country in the world by The Global Gender Gap Index Ranking (Weforum, 2020). According to previous studies, although there are clear sex differences in mate preferences, social change and societal norms may also have an important effect (Bech-Sørensen & Pollet, 2016).

**Figure 1.**
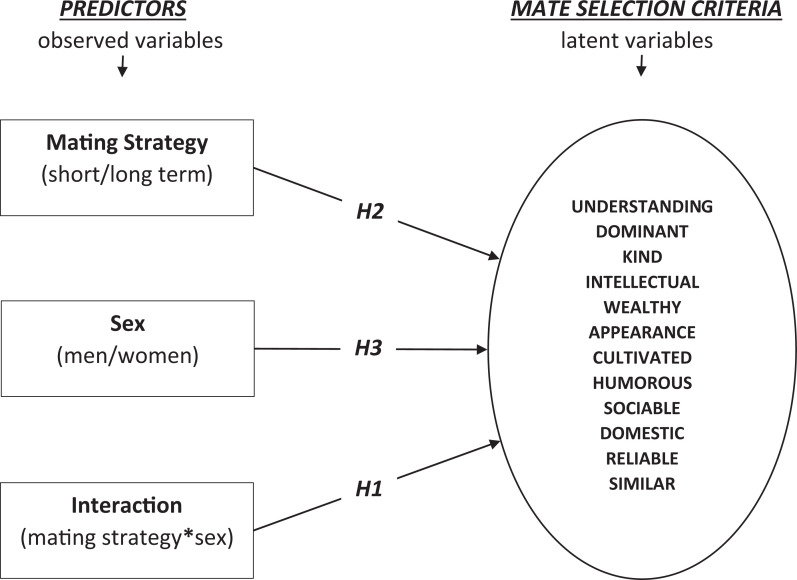
The research model including individual hypotheses.

To summarize the existing literature, men are less selective than women with the exception of appearance. Individuals searching for a short-term partner are less selective than individuals searching for a long-term partner. The expected sex and mating strategy interaction is less clear, i.e. whether or not there are differences in mate preferences of long-term and short-term relationship seekers depending on sex. Based on both the existing literature and evolutionary reasoning, in which men and women differ in their optimal mating strategies in different situations, it would be safe to assume that an interaction effect does exist. Consequently, we hypothesize here and show these hypotheses in Figure 1 that:

*H1*: There are differences in mate selection criteria of long-term and short-term relationship seekers depending on sex (i.e., interaction between mating strategy and sex).
*H2*: There are differences in mate selection criteria of short and long-term relationship seekers (i.e., main effect of mating strategy).
*H3*: There are differences in mate selection criteria of women and men (i.e., main effect of sex).

## Method

### Participants

The necessary data for examining the study’s hypotheses were collected through web survey in October-November 2016 in Norway. The obtained sample (i.e., 1,000) had been stratified according to population percentages of the 19 counties in Norway. Out of these 1,000 respondents 155 indicated that they would like to find a short-term partner (one-night stand or similar) whereas the remaining 845 said that they would like to find a long-term partner (cohabitant, spouse, etc.). There were 417 male and 583 female respondents in the sample.^1^ The sample included 240, 484, and 276 respondents within the age range of 18–34, 35–54, and 55–81 years, respectively. Furthermore, 294 respondents had completed secondary/high school, 321 respondents were studying or completed a bachelor’s degree, and 377 had an education above a bachelor’s degree. Eight of the respondents preferred not to indicate their educational level. Finally, 491 respondents lived in big cities (>100,000 population), whereas 509 respondents lived in smaller cities or rural areas of Norway.

### Measures

In line with the aforementioned hypotheses, this study employed a research model (see Figure 1) that defined the mate selection criteria as the dependent variables, and the mating strategy and sex as the quasi-independent variables. The study adopted a shortened and adjusted list of mate preference criteria developed by Schwarz and Hassebrauck (2012) who generated the following 12 factors using 64 items: Kind and understanding, Dominant, Pleasant, Intellectual, Wealthy and generous, Physically attractive, Cultivated, Humorous, Sociable, Creative and domestic, Reliable, and Similar. Our shortened and adjusted list included 36 items (five of which were excluded due to poor loadings) which resulted in 12 factors listed in Table 1. The respondents (both short- and long-term partner seeking) were asked to indicate how important each of the initial 36 mate preference criteria were using an ordinal scale ranging from 1 (not important at all) to 5 (very important).

## Analysis and Results

The study employed partial least-squares structural equation modeling (PLS-SEM) to estimate the research model, as it was a complex one including a large number of latent variables and indicators (Chin, 2010). Following the algorithm described in Mehmetoglu and Venturini (2021), PLS-SEM estimates factor scores for each case in the sample. Using these scores, structural model parameters (path coefficients) are subsequently estimated using OLS regressions. Prior to testing the hypotheses, the psychometric properties of the measurement model were examined. As shown in Table 1, all of the standardized loadings were very close to or above the suggested threshold of 0.7, AVE values exceeded the recommended level of 0.5, and finally, all the reliability coefficients (D.G. rho) were above the suggested value of 0.7 as well. These findings were indicative of reliability and convergent validity. Further, all of the average variance extracted values were larger than the squared correlations among the latent variables in the model, and thus demonstrated discriminant validity. As the measurement model exhibited evidence of reliability and validity, the structural part (i.e., hypothesis testing) of the model could next be assessed (Henseler et al., 2009).

**Table 1.** Psychometric Properties of the Measurement Model (Loading, Reliability and Communality).

**Latent Variable Manifest Variables**	**Loadings**	**D.G. Rho**	**AVE**
Understanding Considerate Empathic Understanding	0.825 0.835 0.835	0.871	0.692
Dominant Self-confident Goal-oriented	0.776 0.901	0.827	0.706
Kind Kind Helpful	0.773 0.913	0.833	0.715
Intellectual Intelligent Highly-educated Literate	0.812 0.826 0.811	0.857	0.666
Wealthy Rich Has high status Successful	0.805 0.853 0.905	0.891	0.732
Appearance Good looks Sexy Attractive	0.838 0.861 0.762	0.861	0.675
Cultivated Has good manners Polite Well-behaved	0.736 0.876 0.878	0.871	0.694
Humorous Witty Funny Humorous	0.795 0.878 0.840	0.876	0.703
Sociable Outgoing Spontaneous	0.897 0.847	0.864	0.761
Domestic Good at cooking Good at housework	0.612 0.984	0.795	0.671
Reliable Honest Faithful Trustworthy	0.675 0.904 0.781	0.833	0.628
Similar Has similar interests Has similar opinions	0.823 0.889	0.846	0.733

In the first structural part, we tested the interaction effect between mating strategy and sex on all the 12 latent variables (factors) representing the different mate selection criteria (see Table 2).

**Table 2.** The Structural Model (I) With Interaction Effect and Simple Effects (Standardized Coefficients).

Variable	Understanding	Dominant	Kind	Intellectual	Wealthy	Appearance	Cultivated	Humorous	Sociable	Domestic	Reliable	Similar
Longterm	0.148	0.047	0.228	0.069	−0.004	−0.222	0.112	−0.073	−0.139	0.260	0.488	0.119
	(0.001)	(0.299)	(0.000)	(0.125)	(0.925)	(0.000)	(0.012)	(0.112)	(0.002)	(0.000)	(0.000)	(0.009)
Women	0.231	0.161	0.108	0.240	0.110	−0.152	0.231	0.192	0.113	0.117	0.193	0.200
	(0.003)	(0.050)	(0.172)	(0.003)	(0.176)	(0.058)	(0.005)	(0.021)	(0.171)	(0.136)	(0.007)	(0.016)
LongXfem	0.080	0.045	0.094	−0.031	0.143	−0.021	−0.023	−0.030	0.012	0.064	−0.048	−0.074
	(0.354)	(0.620)	(0.284)	(0.736)	(0.115)	(0.812)	(0.796)	(0.745)	(0.893)	(0.465)	(0.550)	(0.418)
r2_a	0.133	0.044	0.115	0.050	0.056	0.090	0.060	0.027	0.025	0.121	0.267	0.030

p-values in parentheses.

That is, we regressed each of these mate selection criteria (e.g., Humorous, Understanding, Sociable etc.) on mating strategy and sex as well as their interaction term. The analysis showed that the interaction effect was not statistically significant on any of the 12 mate selection criteria. In other words, the mate preference criteria differences between short and long-term relationship seekers did not vary depending on sex. As such, our initial hypothesis H1 was not supported. Men and women value similar mate traits both in short- and long-term partners.

In the second structural model, we left out the nonsignificant interaction effect. As such, we regressed the same 12 mate preference criteria on mating strategy and sex alone, the results of which are depicted in Table 3.

**Table 3.** The Structural Model (II) With Main Effects and No Interaction Effect (Standardized Coefficients).

Variable	Understanding	Dominant	Kind	Intellectual	Wealthy	Appearance	Cultivated	Humorous	Sociable	Domestic	Reliable	Similar
Longterm	0.174	0.061	0.260	0.059	0.044	−0.229	0.104	−0.082	−0.135	0.281	0.471	0.094
	(0.000)	(0.067)	(0.000)	(0.077)	(0.182)	(0.000)	(0.002)	(0.015)	(0.000)	(0.000)	(0.000)	(0.005)
Women	0.297	0.198	0.185	0.215	0.228	-0.169	0.212	0.167	0.123	0.169	0.154	0.139
	(0.000)	(0.000)	(0.000)	(0.000)	(0.000)	(0.000)	(0.000)	(0.000)	(0.000)	(0.000)	(0.000)	(0.000)
r2_a	0.133	0.045	0.115	0.052	0.055	0.091	0.061	0.028	0.026	0.121	0.267	0.030

p-values in parentheses.

The results showed that the long-term partner seekers valued the mate criteria of Understanding, Kind, Cultivated, Domestic, Reliable, and Similar statistically significantly more than the short-term partner seekers did. On the other hand, the short-term seekers rated the mate criteria of Appearance, Humorous, and Sociable statistically significantly higher than the long-term partner seekers did. There were no statistical differences found between the short- and long-term partner seekers as far as their consideration of the remaining mate criteria (Dominant, Intellectual, and Wealthy) were concerned. These findings generally supported our second hypothesis, H2.

Moreover, the results showed that the female respondents valued the mate criteria of Understanding, Dominant, Kind, Intellectual, Wealthy, Cultivated, Humorous, Sociable, Domestic, Reliable, and Similar statistically significantly more than the male respondents did. In fact, the only mate criterion the male respondents rated statistically significantly higher than their female counterparts did was Appearance. Our third hypothesis, H3, was also supported by these results.

## Discussion and Conclusion

To recap, we found evidence for sex differences in mate selection criteria: men were more selective with respect to physical attractiveness and women were more selective with respect to all the other mate preference criteria. This was an expected result in light of previous research with similar findings (Buss & Schmitt, 1993; Castro & Lopes, 2011; Regan et al., 2000; Shackelford et al., 2005). The respondents that were searching for a short-term partner had a higher preference for physical attractiveness, humorousness and sociability. The respondents that were searching for long-term relationships were more selective in most of the other mate preference criteria. Perhaps surprisingly, no interaction effect between mating strategy and sex was found. This was contrary to what was predicted, based on Sexual Strategies Theory.

The respondents that were searching for long-term relationships were more selective in most of the other mate preference criteria (see also Castro &Lopes, 2011; Stewart et al., 2000). An issue with previous studies on the topic of short/long term relationship and sex differences is that typically the groups have been analyzed separately while often implying that there is an interaction between the sex and relationship length.

The results also suggested sex differences in preferences depending on the relationship length, but a relationship duration-sex interaction was not explicitly presented (Stewart et al., 2000). Thus, it is not completely clear, whether relationship length and sex interact with each other when they are analyzed together in a single analysis. This is a major question when resolving the hypotheses around this issue.

So, does each sex have also their particular preference when it comes to short-term mating (compared to long-term mating), or do both sexes have the same predictable pattern of preferences? Direct evidence for such an interaction-effect is relatively scarce in general. One exception was a study, in which sex and relationship length had an interaction in which women displayed a higher preference for partner’s sexual passion and desire for short-term partner than long-term partner, whereas there was no such difference among men (Regan et al., 2000). Another study found a sex-relationship length interaction in which both sexes had a similar high preference for attractiveness in short-term relationships but not in long-term relationships, in which women did not pay as much attention to attractiveness (Li & Kenrick, 2006). At least one study found no relationship length-sex interaction and interpreted this as evidence against Sexual Strategies Theory and in favor of Attachment Fertility Theory (Pedersen et al., 2014). Similarly, our results did not support such interaction effect, and thus underlying sex difference in any of the preferences.

One issue that may make interpreting the results more difficult may be the reporting style and underlying choosiness of each sex. For instance, commonly found self-reported preference for physical attractiveness may be influenced by different perception of attraction among different sexes: it is possible that women are more critical in their evaluations.

This study was conducted in an egalitarian, Nordic society, which may be relevant in the study of sex differences in preferences, as they are influenced by social change and societal norms (Bech-Sørensen & Pollet, 2016). Gender equality and strong social safety nets provided by the government may unmask preferences, which might in other environments be hidden under the most urgent materialistic needs. Chinese women, especially those with high socioeconomic status or who lived in cities, preferred “good father” over “good genes” or “good provider” in a self-report study (Lu et al., 2015). Some studies have provided evidence of change in preferences over time (Souza et al., 2016). Studies utilizing personality traits have provided evidence that people prefer traits that are associated to their own traits even in more traditionalistic societies such as Islamic countries (Atari et al., 2020).

Our results lacked the hypothesized interaction-effect, and thus did not support Sexual Strategies Theory, but it is not clear whether or not the results can be interpreted as supporting Attachment Fertility Theory (Pedersen et al., 2014) or some other existing theory. It is also worth remembering that not all traits are adaptations. Some features or traits may be a result of selection for that trait in the other sex (e.g., male nipples) or may otherwise be byproducts of an adaptation (Gould & Lewontin, 1978). It is possible that a similar issue may arise with preferences that are interpreted to be sex-specific or not sex-specific. As an example, it is possible that short-term mate preferences are actually adaptations in men but not in women.

Several studies have studied long- and short-term mating preferences via several different research methods, often in conflicting choice-situation (see Conroy-Beam & Buss, 2019; Cottrell et al., 2007; Mogilski et al., 2019; Perilloux & Cloud, 2019). As their experimental designs and methods differ from the current study, their use as a comparison against the results for this study is not completely straight-forward.

There were some limitations in the sample. The data was self-reported. However, self-reported preference measures are the most commonly used method in other studies of human mate preferences as well. It is also possible that people who are seeking a short-term relationship differ in their attractiveness from the ones who are seeking a long-term relationship. This, in turn, might have an influence on the preferences of the individuals. One final limitation of the study is that for interactions statistical power depends on the number of observations in the smallest cell, which in our case, corresponds to women respondents seeking short-term relationship (n = 63). There were 520 women respondents seeking long-term relationship, 92 men respondents seeking short-term relationship, and 325 men respondents seeking long-term relationship. The number of women seeking short-term relationship was low, as such, power to detect interactions if they exist was low, thus, the non-significant interactions should be interpreted cautiously. Future studies should pay attention the interaction-result that we presented in this study. In ideal case, a large number of women seeking for short-term relationships should be recruited for the study. Perhaps some innovative experimental design could also study this issue in the future.

## Notes

1. Since the two dummy variables “longterm” and “women” were used as predictors in the study’s model, we examined if there could be any multicollinearity issues. The computed tetrachoric correlation of 0.285 was considered too low to cause multicollinearity in the estimation.
